# Atropine

**Published:** 1889-11-16

**Authors:** 


### ATROPINE.

Dr. Tyson, of Folkestone, reports toxic effects from atropine (*British Medical Journal*, October 26th, 1889). A solution, four grains to the ounce, was used for the eyes of a child seven years old, at 9.30 a.m. and at 3 p.m. After the last application the child became giddy and could scarcely walk. She afterwards became quite incoherent, laughing and talking alternately, and thinking she saw "all kinds of things." The symptoms gradually passed off next day. Dr. Tyson remarks that he has seen erysipelatous skin affections of the face follow the application of atropine to the eyes, but not toxic effects, and he thinks the above a most exceptional case. But Dr. Bowles, some years ago, recorded two cases, in a woman and a boy, with alarming symptoms after the application of a few drops of atropine solution to the eyes for ophthalmoscopic purposes. It is known that atropia given to animals may affect the spinal cord, and excite tetanic symptoms. It is also stated to stimulate the vaso-motor centres, and so heighten arterial pressure. It also paralyses the terminations of the oculo-motor nerves, and stimulates the sympathetic so far as the iris is concerned. In short, it seems to have a different action on different parts, for while to a large portion of the central nervous system it acts as a stimulant, to many of the nerves it acts as a paralyser. The symptoms of belladonna poisoning are very similar to those of datura poisoning, and from Dr. Tyson's case it would seem that atropia is capable of exciting similar symptoms. There is not, however, very much known on this subject, for, fortunately, poisoning by the preparations of atropine is rare. It is well, however, to bear in mind that symptoms as mentioned by Dr. Tyson may result from the use of atropine solution to the eyes. Why, we cannot say, and must refer it, somewhat unsatisfactorily, to a peculiar idiosyncrasy of the patient.

